# Risk of Peripheral Arterial Occlusive Disease with Periodontitis and Dental Scaling: A Nationwide Population-Based Cohort Study

**DOI:** 10.3390/ijerph191610057

**Published:** 2022-08-15

**Authors:** Ying-Ting Yeh, Yen-Shuo Tseng, Yi-Liang Wu, Shun-Fa Yang, Bo-Yuan Wang, Yu-Hsun Wang, Liang-Tsai Yeh, Ying-Tung Yeh, Chi-Ho Chan

**Affiliations:** 1 School of Dentistry, Chung Shan Medical University, Taichung 402, Taiwan; 2 Department of Dentistry, Chung Shan Medical University Hospital, Taichung 402, Taiwan; 3 Department of Dermatology, Changhua Christian Hospital, Changhua 500, Taiwan; 4 School of Medicine, Chung Shan Medical University, Taichung 402, Taiwan; 5 Department of Surgery, Chung Shan Medical University Hospital, Taichung 402, Taiwan; 6 Institute of Medicine, Chung Shan Medical University, Taichung 402, Taiwan; 7 Department of Medical Research, Chung Shan Medical University Hospital, Taichung 402, Taiwan; 8 Department of Emergency Medicine, Chung Shan Medical University Hospital, Taichung 402, Taiwan; 9 Department of Anesthesiology, Changhua Christian Hospital, Changhua 500, Taiwan; 10 Department of Post-Baccalaureate Medicine, College of Medicine, National Chung Hsing University, Taichung 402, Taiwan; 11 Department of Microbiology and Immunology, Chung Shan Medical University, Taichung 402, Taiwan

**Keywords:** periodontitis, peripheral arterial occlusive diseases, dental scaling

## Abstract

Periodontitis (PD) is a common oral disease associated with various other diseases, particularly those affecting the cardiovascular system. This study explored whether peripheral artery occlusive disease (PAOD) is associated with PD and dental scaling. This study was a retrospective cohort study design from 2000 to 2018. The study population was newly diagnosed with periodontitis. The comparison group was defined as never diagnosed with periodontitis. The outcome variable was defined with the diagnosis of peripheral arterial occlusive disease (PAOD). The propensity score matching was performed by age, sex, comorbidities, and dental scaling between the two groups. Kaplan–Meier analysis was used to calculate the cumulative incidence of PAOD among the two groups. To perform the independent risk of the PAOD group, the multivariate Cox proportional hazard model was used to estimate the hazard ratios. First, 792,681 patients with PD and 458,521 patients with no history of PD were selected from Taiwan’s Longitudinal Health Insurance Database, which comprises the data of two million beneficiaries. After propensity score matching between the PD and non-PD groups for age, sex, comorbidities, and dental scaling, 357,106 patients in each group were analyzed for PAOD risk. The incidence density, relative risk, and cumulative incidence of PAOD were higher in the PD group than in the non-PD group. After adjusting for all variables, the risk of PAOD for the PD group was greater than for the non-PD group (adjusted hazard ratio = 1.03; 95% CI, 1.01–1.06). Undergoing at least one dental scaling procedure reduced the risk of PAOD. Age over 65 years was also a risk factor. In conclusion, patients with PD have an increased risk of PAOD. In addition, our results can lead to increased attention to oral hygiene, as dental scaling has a trend towards a lower risk of PAOD.

## 1. Introduction

Periodontitis (PD) is one of the most common and influential oral diseases in Taiwan. PD is classified into four stages on the basis of its (i) severity, (ii) complexity of management, (iii) extent, and (iv) distribution [1,2]. The severity of PD is evaluated with several indices such as those for dental plaques, calculus, and pocket depth, bleeding on probing, attachment, and gum loss [3]. Chronic aggressive PD causes destruction of the periodontal ligament, alveolar bone reduction, and subsequent tooth loss [4]. Dozens of species of oral bacteria are associated with PD development. These bacteria have been classified into five complexes according to their relationship with and frequency of detection in PD in decreasing order: red, orange, yellow, green, blue, and purple [5,6]. Three species of bacteria, *Porphyromonas gingivalis*, *Tannerella forsythia*, and *Treponema denticola*, in the red complex are considered the main pathogens responsible for PD and are involved in disease progression and tissue destruction [7]. Other bacteria, such as *Aggregatibacter actinomycetemcomitans*, are the most likely causes of aggressive PD [8]. A retrospective nationwide population study indicated that the prevalence of PD significantly increased over a decade [9]. In addition, PD is common in middle-aged populations and highly prevalent among high school students aged 15 to 18 years [9].

Peripheral arterial occlusive disease (PAOD) is a blood circulation disturbance caused by arterial thrombosis [10]. For example, a recent study indicated that PAOD was associated with a higher risk of heart failure [11]. The pathogenesis of PAOD is similar to that of atherosclerosis, but PAOD always affects the lower limbs. The clinical signs of PAOD are not obvious; people do not notice the disease initially. One of the clinical signs of PAOD is intermittent claudication. Atherosclerosis severity can be determined through ultrasonic measurement of the ankle-brachial index, computed tomography, or magnetic resonance imaging [12,13,14]. PAOD can be divided into four stages based on the severity of symptoms. In stage IV, necrosis, ulcers, and gangrene always occur, even after minor trauma to the toes [15].

PD is associated with cardiovascular diseases. Untreated PD is associated with early atherosclerotic carotid lesions (i.e., increased carotid artery intima-media wall thickness) and higher levels of inflammatory markers (i.e., C-reactive protein and leucocytes) [16]. Male patients with chronic PD have a higher risk of carotid atherosclerosis [17]. Periodontal pathogens may promote atherosclerosis by promoting inflammation and metabolism-related molecular mechanisms [18].

In this study, we enrolled patients with and without PD from the Longitudinal Health Insurance Database 2000 (LHID 2000) and investigated (i) the association between PD and PAOD and (ii) the relationship between dental scaling and PAOD.

## 2. Materials and Methods

### 2.1. Data Source

The LHID is regulated by the Health and Welfare Data Science Center of Taiwan. The database contains 2 million beneficiaries randomly selected from the population of the 2000 beneficiary registry. The database contains all outpatient and inpatient medical claims, including medications, medical operations, procedures, and fees from 2000 to 2018. This study was approved by the Ethics Review Board of Chung Shan Medical University Hospital (CS1-20056).

### 2.2. Study Group and Outcome

This study employed a retrospective cohort study design. Supplementary Table S1 lists the diseases corresponding to the code (International Classification of Diseases, Clinical Modification [ICD-CM]) numbers that define periodontal disease, peripheral arterial occlusive disease, and comorbidities. The study population comprised patients with newly diagnosed PD from 2002 to 2017. Two or more outpatient visits or one or more hospitalizations were necessary to ensure the accuracy of the diagnoses. The index date was considered the first date of a PD code. We excluded patients with PAOD diagnoses from before the index date to confirm new onset. Participants without a PD diagnosis between 2000 and 2018 were also analyzed.

### 2.3. Covariates and Matching

The baseline characteristics considered were age, sex, and hypertension, hyperlipidemia, chronic liver disease, chronic kidney disease, diabetes, chronic obstructive pulmonary disease, rheumatoid arthritis, ankylosing spondylitis, hepatitis B, hepatitis C, herpes zoster, and psoriasis. Two outpatient visits or one hospitalization for the comorbidities were required within 1 year before the index date. In addition, the frequency of dental scaling 1 year before the index date was recorded. In Taiwan, the 65-year-old was defined as the elderly population. We used the cutoff at 65 years.

Age and sex matching in a 1:4 ratio was used to provide an index date for participants with the same starting point. Then, a propensity score matching (PSM) for age, sex, comorbidities, and dental scaling between the 2 groups was performed. The propensity scores were estimated through logistic regression, with the binary variable being PAOD status. PSM helped to account for the heterogeneity of the 2 groups.

### 2.4. Statistical Analysis

PD and non-PD groups were compared using absolute standardized differences. The groups were considered to have similar characteristics when the absolute standardized difference was less than 0.1 [19]. The relative risk (RR) and 95% CIs were calculated using a Poisson regression model. Kaplan–Meier analysis was used to calculate the cumulative incidence of PAOD in the 2 groups. A log-rank test was used to test significance. A Cox proportional hazards model was used to estimate hazard ratios (HRs) for the independent risk of PAOD. The statistical software employed was SAS version 9.4 (SAS Institute, Cary, NC, USA).

## 3. Results

### 3.1. Characteristics of the Participants

**Figure 1:**
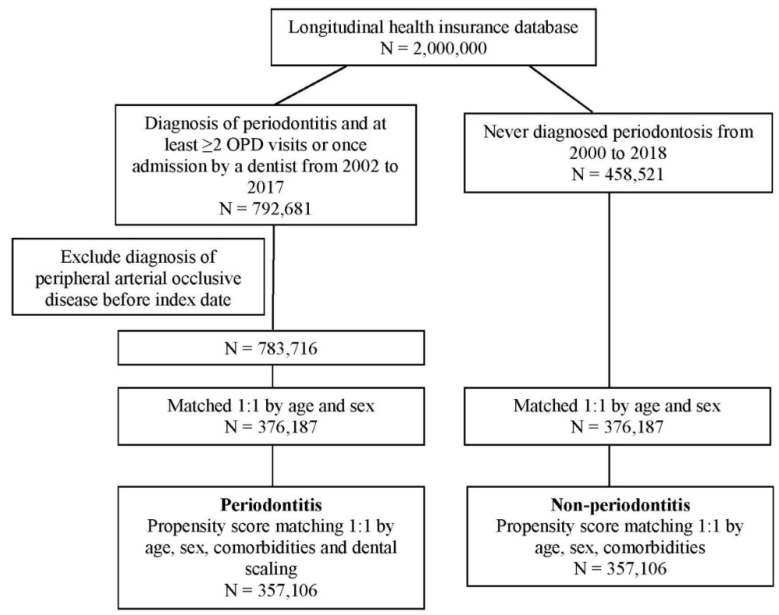
Flowchart of patient selection.

In total, 792,681 patients with PD and 458,521 patients without PD were selected from the LHID. After patients with PAOD before the index date were excluded, 783,716 patients remained in the PD cohort. To evaluate the risk of PAOD by age, sex, comorbidities, and dental scaling in both cohorts, a 1:1 PSM was employed. Finally, 357,106 patients in the PD cohort and the same number of patients without PD in a matched cohort were analyzed for PAOD risk (Figure 1). The demographic characteristics of both study cohorts are presented in Table 1. The mean age in the PD and non-PD groups was 37.56 and 37.78 years, respectively. The majority of patients were male (57%). After PSM, all absolute standardized differences were less than 0.1, suggesting that the age, sex, comorbidities, and frequencies of dental scaling in the groups were similar (Table 1).

### 3.2. Risk of PAOD between PD and Non-PD Group

**Figure 2:**
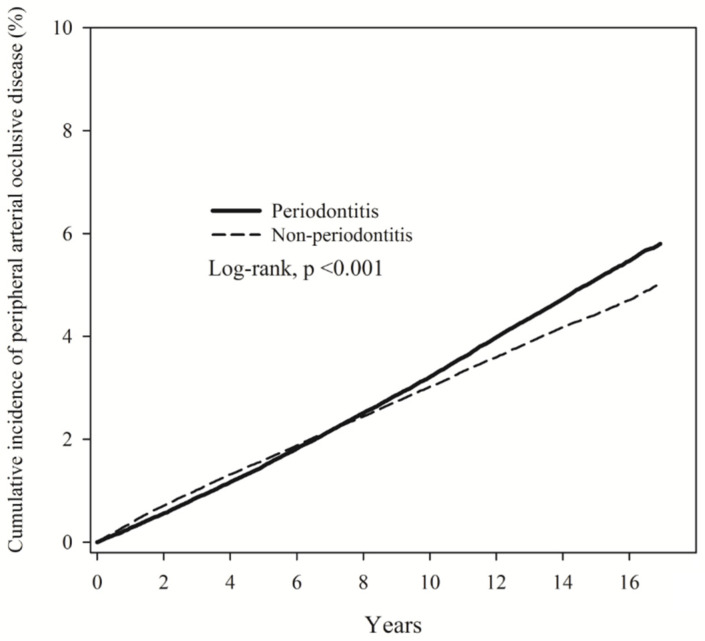
Kaplan–Meier curves of the cumulative proportions of PAOD in periodontitis and non-periodontitis patients.

Poisson regression was employed to compare the RR of PAOD in the PD and non-PD groups. The PD group had a higher PAOD incidence density (3.39) than the non-PD group (ID = 3.09). The RR was 1.11 (95% CI, 1.08–1.13; Table 2). The cumulative incidence of PAOD revealed that the risk of PAOD was higher in the PD group than in the non-PD group (log-rank test, *p* < 0.001; Figure 2).

After adjusting for all variables, the Cox proportional hazards model indicated that the PD group had a higher risk of PAOD than the non-PD group (HR = 1.03; 95% CI, 1.01–1.06) had. The risk of PAOD was also higher among patients 65 years of age (HR = 3.17; 95% CI, 3.07–3.27). Furthermore, comorbidities such as hypertension, hyperlipidemia, chronic liver disease, chronic kidney disease, diabetes, chronic obstructive pulmonary disease, rheumatoid arthritis, ankylosing spondylitis, hepatitis B, hepatitis C, herpes zoster, and psoriasis were risk factors for PAOD. Undergoing one dental scaling procedure was associated with a reduced risk of PAOD (Table 3).

Subgroup analysis revealed that patients aged ≥ 65 had a greater risk of PAOD than those aged < 65 years (*p* = 0.0034) in the PD group. In the PD group, men had a higher risk of PAOD than women had (*p* = 0.0108; Table 4). However, dental scaling was not associated with the risk of PAOD in the PD group (Table 5).

## 4. Discussion

This study analyzed 357,106 patients with PD and an equal number of patients without PD and evaluated the risk of PAOD in both groups after PSM. The PD group had a higher risk of PAOD than the non-PD group. Male patients in the PD group and patients with PD aged older than 65 years had increased risks of PAOD.

The association between PD and the risk of PAOD has been discussed since 1998. Two recent systematic reviews and a prospective population-based cross-sectional cohort study have investigated this association [20,21,22]. Their results indicated that PD could increase the risk of PAOD. The mechanism was considered to be oral bacteria causing thrombosis in the lower limb arteries and provoking an inflammatory response. However, all of these studies lacked information on whether dental scaling could reduce the risk of PAOD in patients with PD. Our study indicated that patients who underwent at least one dental scaling procedure had a lower risk of PAOD.

In the subgroup analysis, patients older than 65 years had a higher risk of PAOD than those younger than 65 years in the PD group. In one cross-sectional analysis, age was demonstrated to be a risk factor, especially for asymptomatic PAOD; other factors such as smoking status, hypertension, and diabetes were strongly associated with PAOD [23]. In addition, a 15% to 20% prevalence of PAOD among individuals aged over 70 years was reported in the United States [15]. Our results are consistent with those of the aforementioned studies.

Despite age, sex disparity was also a risk factor for PAOD. In the subgroup analysis, our results showed that men had a higher risk of PAOD than women had in the PD group. By contrast, a study in the United States demonstrated that women have a higher risk of PAOD [24]. A similar result was also discovered in patients with type 2 diabetes and symptomatic PAOD [25]. These results are probably due to the higher blood concentrations of the C-reactive protein in women than in men. In addition, women have more favorable long-term outcomes than men after percutaneous endovascular revascularization for PAOD treatment [26].

The Cox proportional hazards model revealed that dental scaling reduced the risk of PAOD. However, in the subgroup analysis, the interaction between dental scaling and PAOD in the PD group was not statistically significant, implying that dental scaling might reduce the risk of PAOD in the general population. Dental scaling is a process for dental plaque and calculus removal, but it is not a prescribed therapeutic intervention for PD. However, we did not analyze root planning, periodontal flap surgery, guided tissue regeneration, or other procedures. These therapeutic procedures are typically applied for more severe PD when dental scaling is not completely curative.

Associations between PD and atherosclerotic conditions other than PAOD have been observed over the past decade. For example, the carotid artery intima-media walls were thicker in untreated patients with PD than in patients with PD who received standard treatment [16]. In a nationwide population-based cohort study, male patients with chronic PD had a higher risk of carotid atherosclerosis [17]. Furthermore, PD was also associated with transient ischemic attack and minor ischemic stroke in juveniles [27]. Therefore, a close relationship between PD and PAOD is expected.

The increased risk of PAOD might be due to multiple or indirect mechanisms. In addition to the formation of atherosclerotic lesions in the peripheral arteries due to cholesterol and low-density lipoproteins, other factors promote PAOD. First, patients with PD have long-term inflammation [28]. Several studies have revealed that the blood levels of the C-reactive protein and other inflammatory mediators are strongly associated with PAOD [29,30]. Second, oral bacteremia may be a risk factor for PAOD [18]. Recent studies have identified periodontal pathogen DNA in the atherosclerotic plaques of patients with PAOD [31,32,33]. *P. gingivalis* and *A. actinomycetemcomitans* are the most prevalent periodontal pathogens in atherosclerotic plaques [34]. 

This study has some limitations. First, the LHID does not provide information about the severity of PD, which could affect the risk of PAOD. Second, data on smoking, alcohol consumption, physical activity, and diet were not obtained from the database; such personal behaviors are potential confounders. However, we included related comorbidities and performed PSM to address these factors. Third, the study was a retrospective cohort study; therefore, we could not infer causality.

## 5. Conclusions

Our study demonstrates that PD is associated with PAOD risk, especially in patients older than 65 years. Although no specific interaction of dental scaling with PD affected PAOD risk in the subgroup analysis, dental scaling may generally reduce the risk of PAOD.

## Figures and Tables

**Table 1:** Demographic characteristics of periodontitis and non-periodontitis.

Variables	Before PSM Matching						After PSM Matching					
	Non-Periodontitis (N = 376,187)		Periodontitis (N = 376,187)				Non-Periodontitis (N = 357,106)		Periodontitis (N = 357,106)			
	*n*	%	*n*	%	*p* Value	ASD	*n*	%	*n*	%	*p* Value	ASD
Age					1	0					<0.001	0.029
<20	89,406	23.8	89,406	23.8			89,395	25.0	89,317	25.0		
20–39	114,868	30.5	114,868	30.5			110,646	31.0	110,550	31.0		
40–64	125,667	33.4	125,667	33.4			112,289	31.4	115,839	32.4		
≥65	46,246	12.3	46,246	12.3			44,776	12.5	41,400	11.6		
Mean ± SD	38.27 ± 19.63		38.27 ± 19.63		1	0	37.78 ± 19.80		37.56 ± 19.53		<0.001	0.012
Age					1	0					<0.001	0.029
<65	329,941	87.7	329,941	87.7			312,330	87.5	315,706	88.4		
≥65	46,246	12.3	46,246	12.3			44,776	12.5	41,400	11.6		
Mean ± SD	38.27 ± 19.63		38.27 ± 19.63		1	0	37.78 ± 19.80		37.56 ± 19.53		<0.001	0.012
Sex					1	0					0.0931	0.004
Female	159,735	42.5	159,735	42.5			153,011	42.8	152,309	42.7		
Male	216,452	57.5	216,452	57.5			204,095	57.2	204,797	57.3		
Hypertension	35,598	9.5	41,973	11.2	<0.001	0.056	34,316	9.6	36,249	10.2	<0.001	0.018
Hyperlipidemia	12,091	3.2	18,269	4.9	<0.001	0.084	11,988	3.4	12,559	3.5	0.0002	0.009
Chronic liver disease	9221	2.5	12,203	3.2	<0.001	0.048	9086	2.5	10,125	2.8	<0.001	0.018
Chronic kidney disease	2381	0.6	1657	0.4	<0.001	0.026	1632	0.5	1623	0.5	0.8744	0.000
Diabetes	17,654	4.7	17,941	4.8	0.1191	0.004	15,923	4.5	16,222	4.5	0.0879	0.004
COPD	6740	1.8	7230	1.9	<0.001	0.010	6336	1.8	6534	1.8	0.0782	0.004
Rheumatoid arthritis	829	0.2	950	0.3	0.0041	0.007	794	0.2	798	0.2	0.9201	0.000
Ankylosing spondylitis	315	0.1	503	0.1	<0.001	0.015	315	0.1	294	0.1	0.3946	0.002
Hepatitis B	1775	0.5	2940	0.8	<0.001	0.039	1772	0.5	1945	0.5	0.0044	0.007
Hepatitis C	1164	0.3	1364	0.4	<0.001	0.009	1115	0.3	1182	0.3	0.1614	0.003
Herpes zoster	1272	0.3	1654	0.4	<0.001	0.016	1265	0.4	1418	0.4	0.0031	0.007
Psoriasis	682	0.2	895	0.2	<0.001	0.012	675	0.2	778	0.2	0.0068	0.006
Dental scaling					<0.001	0.157					0.001	0.000
None	353,026	93.8	339,245	90.2			333,945	93.5	334,071	93.5		
1	20,572	5.5	33,320	8.9			20,572	5.8	20,702	5.8		
≥2	2589	0.7	3622	1.0			2589	0.7	2333	0.7		

COPD: Chronic obstructive pulmonary disease.

**Table 2:** Poisson regression of relative risk of PAOD between periodontitis and non-periodontitis.

Variables	Non-Periodontitis	Periodontitis
N	357,106	357,106
Person-years	3,733,623	3,994,111
No. of PAOD	11,450	13,540
ID (95% C.I.)	3.07 (3.01–3.12)	3.39 (3.33–3.45)
Relative risk (95% C.I.)	Reference	1.11 (1.08–1.13)

ID: incidence density (per 1000 person-years).

**Table 3:** Cox proportional hazard model analysis for risk of PAOD.

Variables	Univariable		Multivariable †	
	HR (95% C.I.)	*p* Value	HR (95% C.I.)	*p* Value
Group				
Non-periodontitis	Reference		Reference	
Periodontitis	1.10 (1.08–1.13)	<0.001	1.03 (1.01–1.06)	0.015
Age				
<65	Reference		Reference	
≥65	5.33 (5.20–5.47)	<0.001	3.17 (3.07–3.27)	<0.001
Sex				
Female	Reference		Reference	
Male	0.78 (0.76–0.80)	<0.001	0.87 (0.85–0.89)	<0.001
Hypertension	4.78 (4.65–4.91)	<0.001	1.91 (1.84–1.97)	<0.001
Hyperlipidemia	3.79 (3.64–3.95)	<0.001	1.14 (1.09–1.19)	<0.001
Chronic liver disease	2.49 (2.36–2.62)	<0.001	1.42 (1.34–1.50)	<0.001
Chronic kidney disease	9.73 (8.97–10.55)	<0.001	3.30 (3.04–3.58)	<0.001
Diabetes	5.65 (5.47–5.84)	<0.001	2.22 (2.14–2.31)	<0.001
COPD	3.64 (3.44–3.86)	<0.001	1.40 (1.32–1.48)	<0.001
Rheumatoid arthritis	3.49 (3.01–4.04)	<0.001	1.79 (1.54–2.07)	<0.001
Ankylosing spondylitis	1.86 (1.36–2.55)	<0.001	1.56 (1.14–2.14)	0.005
Hepatitis B	1.65 (1.43–1.91)	<0.001	1.21 (1.04–1.40)	0.011
Hepatitis C	2.93 (2.52–3.40)	<0.001	1.21 (1.04–1.41)	0.015
Herpes zoster	2.51 (2.19–2.88)	<0.001	1.34 (1.17–1.54)	<0.001
Psoriasis	1.82 (1.47–2.25)	<0.001	1.37 (1.11–1.70)	0.004
Dental scaling				
None	Reference		Reference	
1	0.68 (0.64–0.73)	<0.001	0.84 (0.78–0.89)	<0.001
≥2	0.69 (0.57–0.84)	<0.001	0.83 (0.68–1.00)	0.055

COPD: Chronic obstructive pulmonary disease. † Adjusted for all variables.

**Table 4:** Subgroup of Cox proportional hazard model analysis.

Variables	Non-Periodontitis		Periodontitis			
	N	No. of PAOD	N	No. of PAOD	HR (95% C.I.)	*p* Value
Age ^1^						
<65	312,330	7296	315,706	8460	1.00 (0.97–1.04)	0.792
≥65	44,776	4154	41,400	5080	1.04 (1.00–1.08)	0.065
*p* for interaction = 0.0034						
Sex ^1^						
Female	153,011	5885	152,309	6491	0.99 (0.95–1.02)	0.527
Male	204,095	5565	204,797	7049	1.04 (1.00–1.08)	0.042
*p* for interaction = 0.0108						
Hypertension ^1^						
No	322,790	7992	320,857	9449	1.08 (1.05–1.11)	<0.001
Yes	34,316	3458	36,249	4091	0.88 (0.85–0.93)	<0.001
*p* for interaction < 0.001						
Hyperlipidemia ^1^						
No	345,118	10,157	344,547	12,276	1.05 (1.02–1.08)	<0.001
Yes	11,988	1293	12,559	1264	0.75 (0.69–0.81)	<0.001
*p* for interaction < 0.001						
Chronic liver disease ^1^						
No	348,020	10,762	346,981	12,751	1.03 (1.00–1.06)	0.028
Yes	9086	688	10,125	789	0.78 (0.70–0.87)	<0.001
*p* for interaction < 0.001						
Chronic kidney disease ^1^						
No	355,474	11,156	355,483	13,236	1.03 (1.00–1.05)	0.057
Yes	1632	294	1623	304	0.68 (0.58–0.81)	<0.001
*p* for interaction < 0.001						
Diabetes ^1^						
No	341,183	9319	340,884	11,396	1.09 (1.06–1.12)	<0.001
Yes	15,923	2131	16,222	2144	0.72 (0.68–0.76)	<0.001
*p* for interaction < 0.001						
COPD ^1^						
No	350,770	10,931	350,572	12,812	1.02 (0.99–1.04)	0.176
Yes	6336	519	6534	728	0.94 (0.84–1.06)	0.318
*p* for interaction = 0.1053						
Rheumatoid arthritis ^1^						
No	356,312	11,357	356,308	13,456	1.02 (0.99–1.04)	0.199
Yes	794	93	798	84	0.75 (0.56–1.01)	0.059
*p* for interaction = 0.0192						
Ankylosing spondylitis ^2^						
No	356,791	11,431	356,812	13,520	1.02 (0.99–1.04)	0.153
Yes	315	19	294	20	0.89 (0.47–1.69)	0.713
*p* for interaction = 0.7961						
Hepatitis B ^1^						
No	355,334	11,364	355,161	13,442	1.02 (0.99–1.04)	0.217
Yes	1772	86	1945	98	0.83 (0.62–1.12)	0.218
*p* for interaction = 0.1435						
Hepatitis C ^3^						
No	355,991	11,371	355,924	13,448	1.02 (0.99–1.04)	0.233
Yes	1115	79	1182	92	0.82 (0.61–1.11)	0.202
*p* for interaction = 0.2372						
Herpes zoster ^4^						
No	355,841	11,364	355,688	13,421	1.01 (0.99–1.04)	0.269
Yes	1265	86	1418	119	1.04 (0.78–1.37)	0.804
*p* for interaction = 0.9721						
Psoriasis ^3^						
No	356,431	11,407	356,328	13,497	1.02 (0.99–1.04)	0.227
Yes	675	43	778	43	0.73 (0.47–1.11)	0.138
*p* for interaction = 0.081						
Dental scaling ^5^						
None	333,945	11,024	334,071	12,958	1.01 (0.99–1.04)	0.279
1	20,572	379	20,702	526	1.05 (0.92–1.20)	0.454
≥2	2589	47	2333	56	0.78 (0.52–1.16)	0.224
*p* for interaction = 0.296						

^1^ Adjusted for all variables. ^2^ Adjusted for all variables, excluding chronic kidney disease, rheumatoid arthritis, hepatitis C, herpes zoster, and psoriasis. ^3^ Adjusted for all variables, excluding ankylosing spondylitis, and herpes zoster. ^4^ Adjusted for all variables, excluding ankylosing spondylitis, hepatitis C, and psoriasis. ^5^ Adjusted for all variables, excluding rheumatoid arthritis, ankylosing spondylitis, and psoriasis.

**Table 5:** Cox proportional hazard model analysis for risk of PAOD with and without dental scaling.

Variables			Univariable		Multivariable †	
	N	No. of PAOD	HR (95% C.I.)	*p* Value	HR (95% C.I.)	*p* Value
Group						
Non PD Non DS	333,945	11,024	Reference		Reference	
Non PD 1	20,572	379	0.63 (0.57–0.70)	<0.001	0.95 (0.86–1.06)	0.344
Non PD ≥ 2	2589	47	0.70 (0.53–0.94)	0.016	1.13 (0.85–1.51)	0.400
PD Non DS	334,071	12,958	1.10 (1.07–1.13)	<0.001	1.01 (0.99–1.04)	0.300
PD 1	20,702	526	0.79 (0.73–0.87)	<0.001	1.01 (0.93–1.11)	0.777
PD ≥ 2	2333	56	0.75 (0.58–0.98)	0.032	0.88 (0.67–1.14)	0.319
Group						
Non PD Non DS	333,945	11,024	Reference		Reference	
Non PD DS	23,161	426	0.64 (0.58–0.70)	<0.001	0.97 (0.88–1.07)	0.519
PD Non DS	334,071	12,958	1.10 (1.07–1.13)	<0.001	1.01 (0.99–1.04)	0.299
PD DS	23,035	582	0.79 (0.73–0.86)	<0.001	1.00 (0.92–1.09)	0.956

† Adjusted for all variables. Non PD: non-periodontitis. Non DS: non-dental scaling.

## Data Availability

Restrictions apply to the availability of these data. Data were obtained from National Health Insurance database and are available from the authors with the permission of National Health Insurance Administration of Taiwan.

## Supplementary Materials

The following supporting information can be downloaded at: https://www.mdpi.com/article/10.3390/ijerph191610057/s1, Table S1: Disease with relative ICD codes correspond.
