# Supplementary material for: Risk of Peripheral Arterial Occlusive Disease with Periodontitis and Dental Scaling: A Nationwide Population-Based Cohort Study
Source: Int J Environ Res Public Health. 2022 Aug 15;19(16):10057. doi: 10.3390/ijerph191610057 (PMC9408091; doi:10.3390/ijerph191610057)
Supplement: Supplementary file 1 [file ijerph-19-10057-s001.zip › ijerph-1840278-supplementary.pdf]

Supplement Table S1: Disease with relative ICD codes correspond

Disease	International Classification of Diseases, Clinical Modification
Periodontitis	523.4, 523.5, K05.30, K05.31, K05.32, K05.4
Peripheral arterial occlusive disease	443.8, 443.9, 444, I73.81, I73.89, I73.9, I74.01, I74.09, I74.11, I74.2, I74.3, I74.4, I74.5, I74.8, I74.9, I79.1, and I79.8
Hypertension	401-405, and I10-I15
Hyperlipidemia	272, and E78
Chronic liver disease	571, K70, K73, K74, K75.4, K75.81, K76.0, K76.89, and K76.9
Chronic kidney disease	585, N184, N185, N186, and N189
Diabetes	250, E10, E11, and E13
Chronic obstructive pulmonary disease	491, 492, 496, and J41-J44
Rheumatoid arthritis	714.0, M05, and M06
Ankylosing spondylitis	720.0, M45, and M46
Hepatitis B	070.2, 070.3, V02.61, B16.0, B16.1, B16.2, B16.9, B18.0, B18.1, B19.10, 111 B19.11, and Z22.51
Hepatitis C	070.41, 070.44, 070.51, 070.54, 070.7, 112 V02.62, B17.10, B17.11, B18.2, B19.20, B19.21, and Z22.52
Herpes zoster	053, and B02
Psoriasis	696.1, 696.2, 696.3, 696.4, 696.5, L40.0-L40.4, 114 L40.8, L40.9, L41.0, L41.1, L41.3-L41.5, L41.8, L41.9, L94.5, L42, L44.0, and L30.5

* ICD: International Classification of Diseases
